# The Master Transcription Factor *mtfA* Governs Aflatoxin Production, Morphological Development and Pathogenicity in the Fungus *Aspergillus flavus*

**DOI:** 10.3390/toxins8010029

**Published:** 2016-01-20

**Authors:** Zhenhong Zhuang, Jessica M. Lohmar, Timothy Satterlee, Jeffrey W. Cary, Ana M. Calvo

**Affiliations:** 1 Department of Biological Sciences, Northern Illinois University, 155 Castle Dr., Dekalb, IL 60115, USA; xzhzhenhong@163.com (Z.Z.); jlohmar1@niu.edu (J.M.L.); tsatterlee1@niu.edu (T.S.); 2 Food and Feed Safety Research Unit, USDA/ARS, Southern Regional Research Center, New Orleans, LA 70124, USA; jeff.cary@ars.usda.gov

**Keywords:** aflatoxin, *mtfA*, *Aspergillus flavus*, secondary metabolism, conidiation, sclerotia, pathogenicity

## Abstract

*Aspergillus flavus* produces a variety of toxic secondary metabolites; among them, the aflatoxins (AFs) are the most well known. These compounds are highly mutagenic and carcinogenic, particularly AFB_1_. *A. flavus* is capable of colonizing a number of economically-important crops, such as corn, cotton, peanut and tree nuts, and contaminating them with AFs. Molecular genetic studies in *A. flavus* could identify novel gene targets for use in strategies to reduce AF contamination and its adverse impact on food and feed supplies worldwide. In the current study, we investigated the role of the master transcription factor gene *mtfA* in *A. flavus*. Our results revealed that forced overexpression of *mtfA* results in a drastic decrease or elimination of several secondary metabolites, among them AFB_1_. The reduction in AFB_1_ was accompanied by a decrease in *aflR* expression. Furthermore, *mtfA* also regulates development; conidiation was influenced differently by this gene depending on the type of colonized substrate. In addition to its effect on conidiation, *mtfA* is necessary for the normal maturation of sclerotia. Importantly, *mtfA* positively affects the pathogenicity of *A. flavus* when colonizing peanut seeds. AF production in colonized seeds was decreased in the deletion *mtfA* strain and particularly in the overexpression strain, where only trace amounts were detected. Interestingly, a more rapid colonization of the seed tissue occurred when *mtfA* was overexpressed, coinciding with an increase in lipase activity and faster maceration of the oily part of the seed.

## 1. Introduction

Species of the genus *Aspergillus* produce a wide variety of secondary metabolites. Some of these compounds have beneficial bioactive properties, such as antibiotic or anticholesteremic, while others are deleterious, such as mycotoxins [1]. Some of these fungal species are opportunistic plant pathogens. Among them, *A. flavus* is capable of contaminating agricultural products, particularly oil seeds, at pre-harvest and post-harvest [2]. This is particularly relevant, since *A. flavus* is notorious for producing highly toxic and carcinogenic mycotoxins. The most well-known are the polyketide-derived compounds known as aflatoxins (AFs), especially AFB_1_, the most carcinogenic natural compound identified. Ingestion of aflatoxin-contaminated food can result in hepatotoxicity, liver cancer, teratogenicity, immunotoxicity and death [3,4,5,6,7]. Searching for novel genetic targets could lead to new strategies to decrease the negative impact of aflatoxin contamination of food supplies.

Considerable progress has been made in the elucidation of the genetic regulatory networks involved in the control of AF production in *A. flavus* (*i.e.*, [8,9,10,11,12,13]). The study of mycotoxin production in the model *fungus A. nidulans* has greatly contributed to accelerating the pace in discovering AF regulators in *A. flavus. A. nidulans* produces sterigmatocystin (ST), the penultimate precursor in the AF biosynthetic pathway. Both clusters contain *aflR* homologs, encoding a Zn(II)_2_Cys_6_ transcription factor required for the activation of these gene clusters [14,15,16,17,18,19]. Recent research using this model organism revealed a new regulatory gene, *mtfA*, encoding a C_2_H_2_ zinc finger transcription factor that affects ST production. *mtfA* was originally identified through a mutagenesis screening technique designed to reveal novel *veA*-dependent elements involved in ST regulation in *A. nidulans* [20]. Both deletion and overexpression of *mtfA* lead to a reduction of ST production compared to the wild-type strain. Additional analyses showed a broader regulatory role of *mtfA* in *A. nidulans*, controlling genes involved in the production of other secondary metabolites, besides those involved in ST production [20,21]. *mtfA* was also identified as a genetic link between secondary metabolism and morphogenesis, positively affecting both asexual and sexual development in the model fungus [20]. Furthermore, this master regulator was also found to perform similar roles in *A. fumigatus*, an opportunistic animal pathogen that is the most frequent cause of aspergillosis in immunocompromised patients [22]. Absence of *mtfA* in *A. fumigatus* resulted in alteration in the production of secondary metabolites and conidiation, as well as in a reduction in virulence, as shown in the *Galleria mellonella* animal model for invasive aspergillosis [22].

A recent transcriptome analysis of *mtfA* in *A. nidulans* and *A. fumigatus* demonstrated the importance of this regulatory gene as a keystone in the control of the expression of hundreds of genes in these two fungi, extensively affecting secondary metabolite gene clusters. This regulatory scope includes clusters involved in the synthesis of mycotoxins [21], some of them known to be virulence factors during infection (*i.e.*, [23,24,25,26,27]). Genomic analysis indicated that *mtfA* is conserved in numerous filamentous fungi, particularly among Ascomycetes [20], and it was not found in plant or animal genomes, suggesting that *mtfA* could be a potential target to control the detrimental effects of numerous pathogenic fungi. In this study, we investigated *mtfA* in *A. flavus*, revealing a regulatory role in AF production, development and pathogenicity of this agriculturally- and medically-important fungus.

## 2. Materials and Methods

### 2.1. Fungal Strains and Growth Conditions

*Aspergillus flavus* strains used in the study are listed in Table 1. The strains were cultured on YGT (0.5% yeast extract, 2% glucose and trace elements, as described in [10]), unless otherwise indicated. The medium was supplemented as required depending on the presence of auxotrophic markers [10,28]. Solid media were prepared by adding 10 g/L of agar, except top YGT agar medium (5 g/L). Strains were stored in 30% glycerol at −80 °C.

**Table 1:** *Aspergillus flavus* strains used in this study.

Strain Name	Related Genotype	Source
CA14	*pyrG*^−^, *niaD*^−^, Δ*ku70*	[29]
CA14-*pyrG*-1	*niaD*^−^, Δ*ku70*	[29]
CA14 *pyrG*-*1*-*niaD^+^*	prototroph, Δ*ku70*	This study
TZZ1	Δ*mtfA*::*pyrG. niaD*^−^, Δ*ku70*	This study
TZZ2	Δ*mtfA::pyrG*, Δ*ku70*	This study
TZZ3	Δ*mtfA::pyrG. niaD*^−^, *mtfA*::*niaD*^+^, Δ*ku70*	This study
TZZ4	*gpdA*(p)::*mtfA::trpC*(t)::*pyrG. niaD*^−^, Δ*ku70*	This study
TZZ5	*gpdA*(p)::*mtfA::trpC*(t)::*pyrG*, Δ*ku70*	This study

### 2.2. Generation of the Deletion, Complementation and Overexpression mtfA Strains

The *mtfA* deletion cassette was constructed by fusion PCR as previously described [30]. Both 1.4-kb and flanking untranslated regions (5′UTR and 3′UTR) were PCR amplified from *A. flavus* genomic DNA with primer sets *mtfA*-del-1 and *mtfA*-del-2 and *mtfA*-del-3 and *mtfA*-del-4 respectively (Table 2). The intermediate fragment containing the *A. fumigatus pyrG* marker was amplified from plasmid p1439 [31] by primers *mtfA*-del-5 and -6 (Table 2). The three fragments were PCR fused using primers *mtfA*-del-1 and *mtfA*-del-4. Polyethylene glycol-mediated transformation of *A. flavus* CA14 (Δ*ku70. pyrG*-, *niaD*-) protoplasts was carried out as described by Cary *et al.* [32], resulting in the generation of the *mtfA* deletion strain, TZZ1 (Δ*ku70*, Δ*mtfA. niaD*-). Replacement of the entire *mtfA* coding region with *pyrG* was confirmed by Southern analysis, as previously described [33]. A prototroph of this deletion strain, TZZ2 (Δ*ku70*, Δ*mtfA*), was obtained by a second transformation with the *niaD* wild-type allele from *A. fumigatus* (Table 1).

**Table 2:** Primers used in this study.

Primer Name	Sequence
*mtfA*-del-1	CCCCCATGATTAATGATTGATGGATTTCTGGGCG
*mtfA*-del-2	GGGAGAGCTTGAGCTGTGGAAGGTGGAAGGAT
*mtfA*-del-3	ACCAAAGCACAAAGACAAGAAACTAAAA
*mtfA*-del-4	TACATATGGCATCCTCTCACGAACGTC
*mtfA*-del-5	ATCCTTCCACCTTCCACAGCTCAAGCTCTCCCGCCTCAAACAATGCTCTTCACCC
*mtfA*-del-6	TTTTAGTTTCTTGTCTTTGTGCTTTGGTGTCTGAGAGGAGGCACTGATGC
C-NsiI-S	NNNNNNNATGCATGATTCATCCCCCATGATTAA
C-Nsil-A	NNNNNNNATGCATTACATATGGCATCCTCTCAC
*mtfA*-S	GATTCATCCCCCATGATTAA
*mtfA*-A	TACATATGGCATCCTCTCAC
O-AscI-S	AAAAAGGCGCGCCATGGATCTCGCCAGCCTTATCACTCC
O-NotI-A	AAAAAAAGCGGCCGCTTATACCATGGCGGTGGCGACG
gpdA-p	AAGTACTTTGCTACATCCATACTCC
niaD-S	ACCGGTCGCCTCAAACAATGCTCTGGCAATGTGAGGCTCCTCCCCAATC
niaD-A	GTCTGAGAGGAGGCACTGATGCGGCGATCTCTGGATCAATACGACCGAC
qPCR-Afla_18S_F	TGATGACCCGCTCGGCACCTTACGAGAAATCAAAGT
qPCR-Afla_18S_R	GGCCATGCACCACCATCCAAAAGATCAAGAAAGAGC
qPCR-Afla_ver1_F	GCGGAGAAAGTGGTTGAACAGATC
qPCR-Afla_ver1_R	CAGCGAACAAAGGTGTCAATAGCC
qPCR-Afla_brlA_F	TATCCAGACATTCAAGACGCACAG
qPCR-Afla_brlA_R	GATAATAGAGGGCAAGTTCTCCAAAG
qPCR-Afla_aflR_F	GCAACCTGATGACGACTGATATGG
qPCR-Afla_aflR_R	TGCCAGCACCTTGAGAACGATAAG
qPCR-Afla_*mtfA*_F	AGTGTGGCCTCGTACTCTTCGCCGGTTGAATCCTC
qPCR_Afla_*mtfA*_R	GTCGTGGTTCTGTTGGTAGGGTGCCGAGCTGGAAG

The *mtfA* complementation strain was constructed by transforming the *mtfA* deletion mutant with the *mtfA* wild-type allele. The complementation *mtfA* vector was generated as follows: a DNA fragment containing the coding region of *mtfA* and 1.4 kb 5′ and 3′UTRs was PCR amplified with primers C-NsiI-S and C-NsiI-A (Table 2) and digested by *NsiI*. The digested PCR fragment was cloned into pSD52 plasmid previously digested with the same enzyme. pSD52 harbored the selection marker *niaD* from *A. fumigatus*. The resulting recombinant plasmid, pSD52.2-*mtfA*, was used to transform TZZ1 (Δ*ku70*, Δ*mtfA. niaD*-), obtaining the *mtfA* complementation prototroph TZZ3 (Table 1). The presence of pSD50.2-*mtfA* in TZZ3 was confirmed by diagnostic PCR using primers *mtfA*-del-1 and *mtfA*-del-4.

In order to obtain the *mtfA* overexpression strain (OE*mtfA*), a fragment containing the *mtfA* coding region was PCR amplified from CA14 (Table 1) genomic DNA with primers O-AscI-S and O-NotI-A (Table 2). The fragment was digested with *Asc*I and *Not*I and ligated into pTDS1 previously digested with the same enzymes. pTDS1 contains the *A. nidulans gpdA* promoter and *trpC* terminator. The resulting vector, pTDS1-*mtfA*, was then transformed in CA14, generating TZZ4 (Table 1). Integration of pTDS1-*mtfA* into TZZ4 was verified by diagnostic PCR with primers gpdAp and O-Not1-A. TZZ4 was then transformed with the wild-type allele of *niaD* from *A. fumigatus*, obtaining the prototroph TZZ5 (Table 1).

### 2.3. Aflatoxin B_1_ Analysis

AFB_1_ was extracted with chloroform from fungal cultures grown as specified in each case. Extracts were allowed to dry and then resuspended in 500 μL of CHCl_3_ before 25 μL of each extract was fractionated on a silica gel thin-layer chromatography (TLC) using a toluene-ethyl acetate-formic acid (5:4:1, *v*/*v*/*v*) solvent system. TLC plates were sprayed with AlCl_3_ (15% in ethanol) and baked at 80 °C for 10 min to intensify AFB_1_ fluorescence upon exposure to long-wave UV fluorescence at 375 nm. Commercial AFB_1_ (Sigma-Aldrich, St. Louis, MO, USA) was used as the standard.

### 2.4. Morphological Analysis

Conidia (10^6^ spores/mL) of *Aspergillus flavus* wild-type (CA14 *pyrG*-*1*-*niaD+*), Δ*mtfA*, (TZZ2) complementation (TZZ3) and OE*mtfA* (TZZ5) strains were added to 5 mL of top-agar (0.4%) that was subsequently poured onto the surface of YGT agar medium. Seven-millimeter diameter cores were collected during a time-course experiment. The cores were homogenized, and conidia were quantified with a hemocytometer (Hausser Scientific, Horsham, PA, USA) under a Nikon Eclipse E-400 bright-field microscope (Nikon Inc., Melville, NY, USA).

The effect of *mtfA* on sclerotial production was also assessed under the same experimental conditions. Sixteen millimeter cores were sprayed with 70% EtOH in order to improve visualization of sclerotia. Micrographs were taken using a Leica MZ75 dissecting microscope coupled to a Leica DC50LP camera (Leica Microsystems Inc., Buffalo Grove, IL, USA).

### 2.5. Gene Expression Analysis

Strains were incubated in liquid stationary cultures in Petri dishes containing 30 mL of liquid YGT. Plates inoculated with conidia (10^6^ spores/mL) of *A. flavus* wild-type, Δ*mtfA*, complementation and OE*mtfA* strains were incubated at 30 °C in the dark. Total RNA was extracted from lyophilized mycelia using TRIsure (Bioline, Taunton, MA, USA) reagent and an RNeasy Plant Mini Kit (Qiagen, Valencia, CA, USA) according to the manufacturer instructions. Gene expression analysis was performed by qRT-PCR. First, five micrograms of total RNA were treated with RQ1 RNase-Free Dnase (Promega, Madison, WI, USA). cDNA was synthesized with Moloney murine leukemia virus (MMLV) reverse transcriptase (Promega). qRT-PCR was performed with the Applied Biosystems 7000 Real-Time PCR System using SYBR green dye for fluorescence detection. *A. flavus* 18S ribosomal gene expression was used as the reference, and the relative expression levels were calculated using the 2^−ΔΔCT^ method [34]. The primer pairs used are indicated in Table 2.

### 2.6. Pathogenicity Study

The NC94022 Virginia peanut line was utilized to examine the possible role of *mtfA* on seed infection as previously described [13] with minor modifications. All seeds used in this experiment were shelled, separated, the embryos removed and weighed out to approximately 0.25 g to 0.35 g. Each cotyledon was surface sterilized with 10% Clorox bleach for approximately 1 min and then washed with sterile ddH_2_O twice. The viable cotyledons were then dried and placed on sterile glass Petri dish plate (12 peanut cotyledons per plate). Individual cotyledons were inoculated on the adaxial surface with 50 µL of water containing approximately 2.0 × 10^6^ spores/mL. The cultures were incubated for 7 days at 30 °C in the dark.

Ergosterol content is used as an indicator of fungal burden in infected seeds [13,17]. After 4 and 7 days of incubation at 30 °C, four infected peanut cotyledons were collected from each culture, ground in liquid nitrogen and extracted with a 4-mL solution of chloroform-methanol in a 2:1 ratio overnight at room temperature. The extraction mixtures were then filtered through sterile Miracloth into a 50-mL beaker. The extracts were allowed to evaporate and re-suspended in 3 mL of the same extraction mixture. One milliliter of each sample was filtered through a 0.2-micron filter and placed into a 1-mL HPLC vial for analysis. Twenty-five microliters of each sample were injected into a Waters 1525 HPLC system (Waters, Milford, MA, USA) equipped with a binary pump and a Waters 717 autosampler (Waters, Milford, MA, USA). HPLC separation was carried out at 50 °C on a Phenomenex C18 4.6 × 25 mm 5-micron analytical column attached to a column guard and 100% HPLC grade methanol as the mobile phase at a flow rate of 1.0 mL/min. UV detection at 282 nm was performed with a Waters 2487 Dual λ Absorbance Detector (Waters, Milford, MA, USA). HPLC-grade ergosterol (Sigma-Aldrich, St. Louis, MO, USA) was used as standard reference to determine ergosterol concentration in the samples. The experiment was carried out with 3 replicates.

AFB_1_ content in infected peanut cotyledons was evaluated after 7 days of incubation at 30 °C. Four cotyledons were collected from each culture and ground in liquid nitrogen. Then, 12 mL of sterile ddH_2_O were added, and the suspensions were placed in sterile 50-mL beakers to which 6 mL of acetone was added. The beakers were then placed on a rotary platform. After 1 h, each sample was filtered through Whatman paper and placed in a 50-mL Falcon tube to which 17 mL of methylene chloride were added. The content of each tube was mixed by inversion and centrifuged at 4000 rpm for 5 min. The bottom organic layer was collected, passed through a filter with granulated sodium sulfate to absorb remaining water and allowed to evaporate overnight. The dried extracts were re-suspended in 300 µL of acetone and transferred into 1.5-mL Eppendorf tubes. The extracts were again allowed to evaporate overnight and re-suspended in 100 μL of acetone. Twenty-five microliters of each extract were then separated by TLC as described above. This experiment was carried out in triplicate.

Conidial production on the infected seeds was examined after 7 days of incubation at 30 °C in the dark. In this case, four cotyledons were harvested from each culture and placed into 1.5-mL Eppendorf tubes. One milliliter of ddH_2_O was added to each tube and vortexed for 1 min. Conidia were quantified with a hemocytometer (Hausser Scientific, Horsham, PA, USA) as detailed above.

### 2.7. Hydrolytic Activity Analysis

*Aspergillus flavus* produces a variety of extracellular hydrolytic enzymes required for successful colonization and subsequent toxin contamination of crops [35,36]. For this reason, we also investigated whether *mtfA* affects hydrolytic activity in *A. flavus*, specifically lipase, protease and amylase activity.

#### 2.7.1. Lipase Activity

Lipase activity was examined as previously described [37] with minor modifications. Briefly, 100 μL of conidial suspensions (10^6^ spores/mL) of the *A. flavus* wild-type, Δ*mtfA*, complementation and OE*mtfA* strains were inoculated on 10 mL of tributyrin agar (per liter: 3 g yeast extract, 5 g peptone, 10 mL tributyrin, 10 g agar, pH 7.5) in test tubes with 6 replicates per strain. The inoculated cultures were incubated at 30 °C in the dark. Zones of degradation were measured after 4 to 7 days of incubation.

#### 2.7.2. Protease Activity

To assess whether *mtfA* plays a role influencing protease activity in *A. flavus*, the strains (10^6^ spores/mL) were inoculated into 500 mL of PMS (Peptone Minimal Salts) broth (per 1 L: 50 g peptone, 3 g (NH_4_)_2_SO_4_, 10 g K_2_HPO_4_, 2 g MgSO_4_∙7 H_2_O, 1 mL of trace elements, pH 5.2) and incubated at 37 °C at 250 rpm for 24 h. To induce protease activity, approximately 1 g of mycelia was shifted into 25 mL of liquid 0.01% GMM (Glucose Minimum Medium) containing 8 mg/mL of bovine serum albumin (BSA) and allowed to further incubate at 250 rpm at 30 °C. Fungal supernatants were collected after 24, 48 and 72 h of incubation and filtered through 0.2-μm low-protein-binding filters. Protease activity was measured by an azocasein assay as previously described [38].

#### 2.7.3. Amylase Activity

Amylase activity was evaluated by TLC as described by Duran *et al.* [38] with some modifications. The strains were inoculated in a 1-L flask (10^6^ spores/mL) containing 500 mL PMS broth. The cultures were incubated at 37 °C for 24 h at 250 rpm. After incubation, mycelia were washed three times with sterile ddH_2_O before shifting approximately 1 g of mycelia into a 125-mL flask containing 25 mL of an amylase-inducing medium (GMM, with 1% starch as the carbon source instead of glucose). The cultures were incubated at 30 °C at 250 rpm. After 24, 48 and 72 h of incubation, approximately 100 μL of fungal supernatant were mixed with 100 μL of 0.5% maltoheptose solution and incubated at 40 °C for 18 h. The reaction was stopped by heating the mixture to 100 °C for 5 min. Five microliters of the degradation products were loaded onto a Silica Pre-Coated Polygram Sil G/UV_254_ TLC plate (Macherey-Nagel, Bethlehem, PA, USA) and compared to 5 μL of glucose, maltose and maltotriose standards (1 mg/mL). The TLC plate was then developed in an isopropanol-water-ammonium hydroxide (70:30:10, *v*/*v*) solvent system. After development, the TLC plate was sprayed with 30% sulfuric acid and dried before being charred at 100 °C for 5 min and photographed with a Sony Cybershot DSC-W120 camera (Sony, New York, NY, USA).

### 2.8. Statistical Analysis

Quantitative data were analyzed using ANOVA (analysis of variance) in conjunction with Tukey’s test. Differences among mean comparisons of the *A. flavus* strains were considered significant if the *p*-value was less than 0.05 (*p* < 0.05).

## 3. Results

### 3.1. mtfA Affects AFB_1_ Biosynthesis as well as the Production of Other Unknown Secondary Metabolites in A. flavus

**Figure 1:**
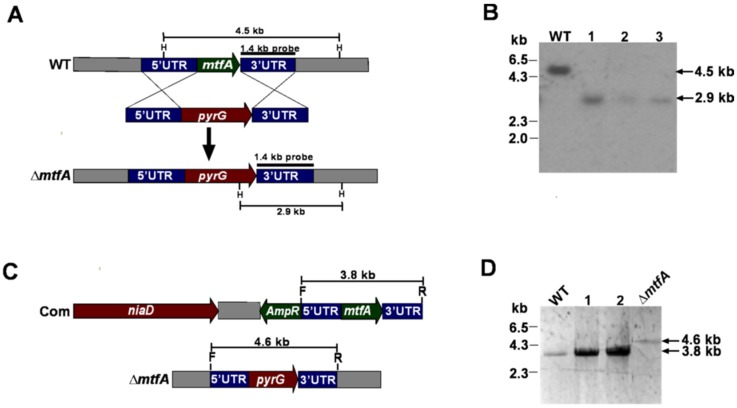

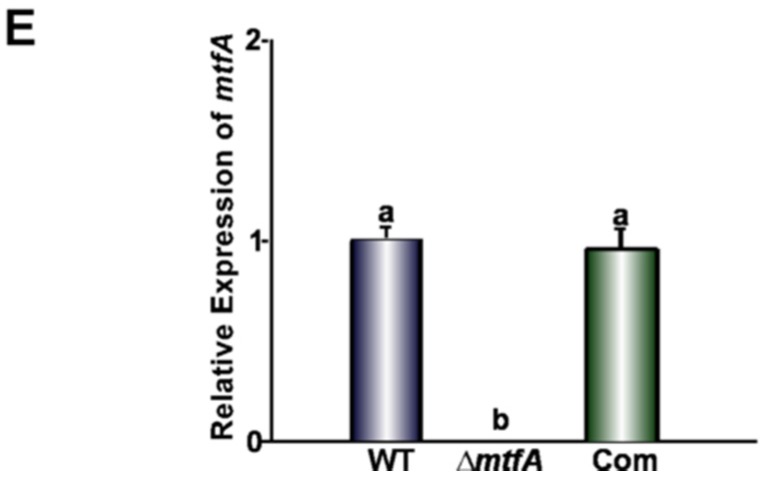
Generation and verification of Δ*mtfA* and *mtfA* complementation strains. (**A**) Diagram showing the replacement of *mtfA* with the *pyrG* marker by a double-crossover event. HindIII restrictions sites and the probe template are shown. (**B**) X-ray image of the Southern blot analysis confirming the deletion of *mtfA*. Genomic DNA samples were digested with HindIII (H). The Expected band sizes were 4.5 kb for the wild-type and 2.9 kb for Δ*mtfA*. All mutant strains presented the same phenotype; transformant 1 (TZZ2) was used for further studies. (**C**) Linearized representation of the complementation plasmid (pSD2.2-*mtfA*). (**D**) Results of diagnostic PCR, confirming the insertion of the complementation plasmid carrying the *mtfA* wild-type allele in the Δ*mtfA* strain, using primers C-NsiI-S and C-NsiI-A (Table 2), indicated as F and R, respectively in (**C**). The wild-type strain and deletion *mtfA* mutant were used as controls. The presence of a 3.8-kb band shows proper integration in the *mtfA* locus, while a 4.6-kb band indicates the altered locus in Δ*mtfA*. (**E**) Expression analysis of *mtfA* by qRT-PCR with primers qPCR-Afla_*mtfA*_F and qPCR-Afla_*mtfA*_R (Table 2). The relative expression was calculated using the 2^−ΔΔCT^ method, as described by Livak and Schmittgen [34]. The expression of 18S rRNA was used as an internal reference. Values were normalized to the expression levels in the wild-type, considered as one. Error bars represent the standard errors. Different letters above the bars represent significantly different values (*p* ≤ 0.05).

**Figure 2:**
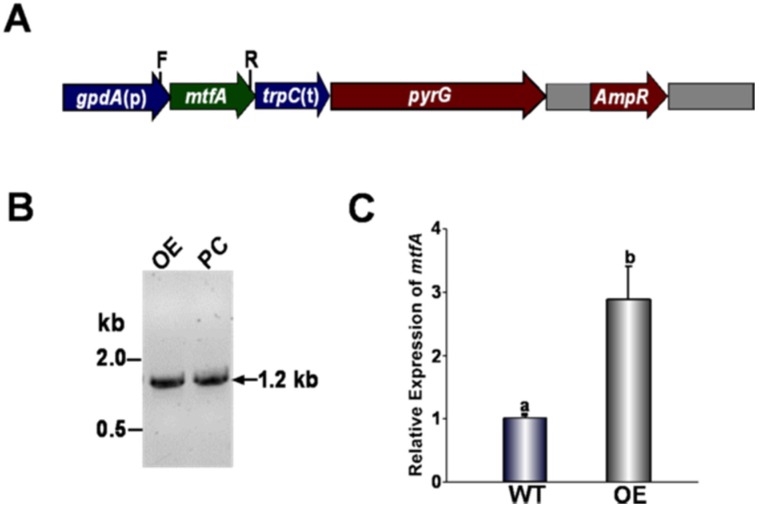
Generation and verification of *mtfA* overexpression strain (OE*mtfA*). (**A**) Linearized representation of the pTDS1-*mtfA* plasmid. Primers gpdA-p and O-NotI-A, shown as F and R, respectively, were used for PCR amplification. (**B**) A 1.2-kb PCR product indicating the integration of the overexpression plasmid into the genome. The overexpression plasmid was used as a positive control (PC). (**C**) Expression analysis of *mtfA* by qRT-PCR indicating greater accumulation of *mtfA* transcripts in the *mtfA* overexpression strain compared to that of the wild-type. The relative expression was calculated using the 2^−ΔΔCT^ method, as described by Livak and Schmittgen [34]. Expression of 18S rRNA was used as an internal reference. Values were normalized to the expression levels in the wild-type, considered as one. Error bars represent the standard errors. Different letters above the bars represent significantly different values (*p* ≤ 0.05).

In order to elucidate the role of *mtfA* (Accession Number AFLA_091490) in *A. flavus* wild-type, deletion (Δ*mtfA*) (TZZ2), complementation (TZZ3) and overexpression (OE*mtfA*) (TZZ5) strains were generated as specified in the Materials and Methods Section. The strains were verified by Southern blot analysis or diagnostic PCR (Figure 1 and Figure 2). In addition, *mtfA* expression levels in wild-type, Δ*mtfA*, complementation and OE*mtfA* strains were also examined (Figure 1E and Figure 2C). As predicted, the Δ*mtfA* strain did not show *mtfA* expression. Expression of *mtfA* in the OE*mtfA* strain was greater than in the wild-type strain, indicating that the overexpression cassette was functional.

**Figure 3:**
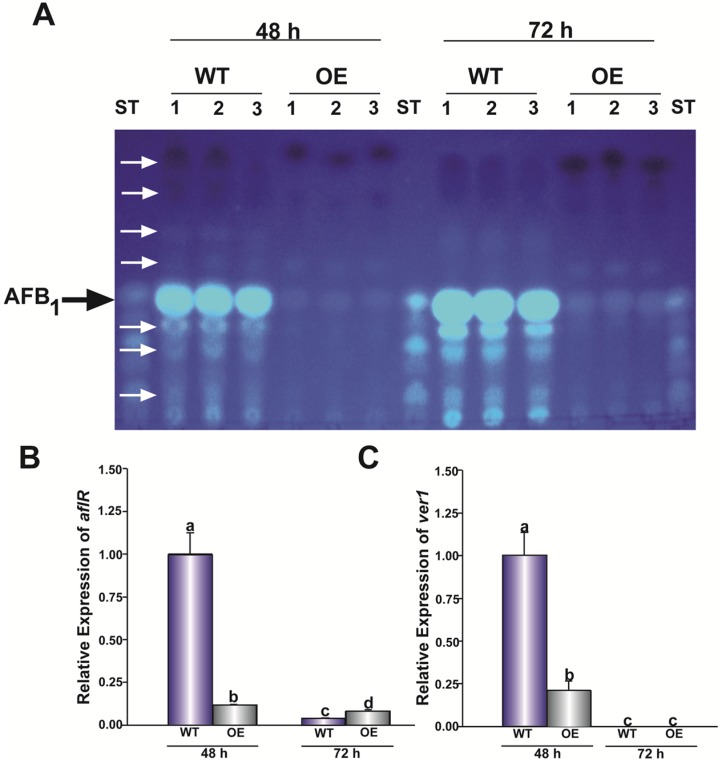
Role of *mtfA* in AFB_1_ production. (**A**) TLC analysis of YGT liquid stationary cultures of wild-type (WT) CA14 *pyrG*-*1*-*niaD+* and overexpression strain (OE) (TZZ5) after 48 h and 72 h of incubation. Unknown metabolites are indicated by white arrows. ST (sterigmatocystin) indicates the AFB_1_ standard. Expression analysis of *aflR* (**B**) and *ver1* (**C**). The relative expression was calculated using the 2^−ΔΔCT^ method, as described by Livak and Schmittgen [34]. The expression of 18S rRNA was used as an internal reference. Values were normalized to the expression levels in the wild-type, considered as one. Error bars represent the standard errors. Different letters above the bars represent significantly different values (*p* ≤ 0.05).

*mtfA* was previously shown to regulate the production of several secondary metabolites, including ST, penicillin and terriquinone A in *A. nidulans* [20] and gliotoxin in *A. fumigatus* [22]. Furthermore, transcriptome analyses show a broad effect of *mtfA* affecting many genes in the genome of these two fungi [21]. To assess whether *mtfA* was necessary for *A. flavus* secondary metabolism, we specifically examine its possible role in the regulation of AFB_1_ biosynthesis. Our TLC analyses revealed that overexpression of *mtfA* dramatically reduces AFB_1_ production compared to the control strains (Figure 3A). The observed reduction was accompanied by a drastic reduction in *aflR* expression, as well as a reduction in *ver*-1 expression, commonly used as an indicator of AF cluster activation (Figure 3B,C). The absence of the *mtfA* did not have a significant effect on AFB_1_ production under the conditions tested (data not shown). It was also noted by TLC analysis that other unknown metabolites were absent or produced at lower levels in the OE*mtfA* strain, while they were present or produced at higher amounts in the wild-type strain (Figure 3A).

### 3.2. Morphological Development Is Regulated by mtfA in A. flavus

**Figure 4:**
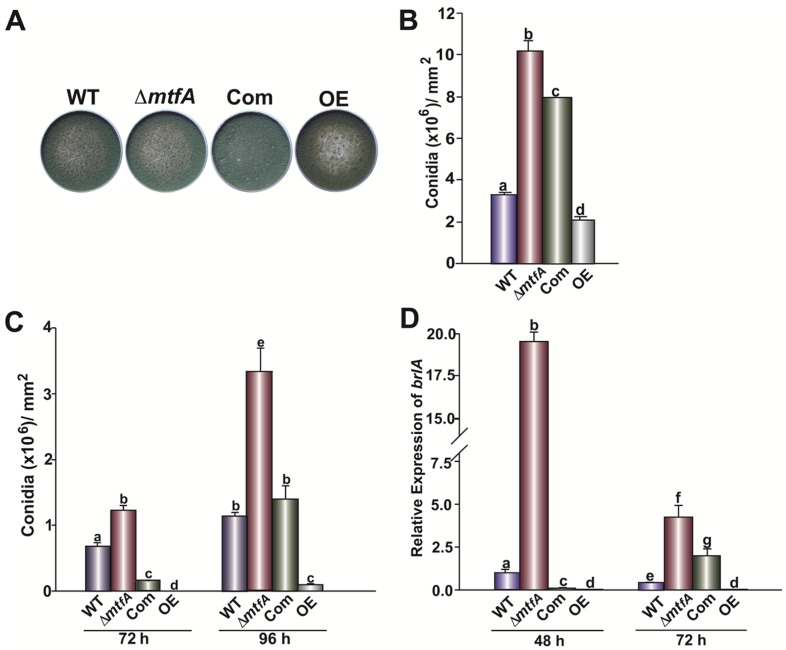
*mtfA* negatively regulates *A. flavus* asexual development. (**A**) Photographs of YGT top-agar inoculated cultures of *A. flavus* wild-type (WT), Δ*mtfA*, complementation (Com) and *mtfA* overexpression (OE). Strains were incubated for seven days at 30 °C. (**B**) Conidial quantifications of cultures shown in (**A**). (**C**) Conidial quantifications of liquid stationary cultures after 48 and 72 h of incubation. (**D**) Relative expression levels of *brlA*. The error bars represent the standard errors. Different letters above the bars represent significantly different values (*p* ≤ 0.05).

**Figure 5:**
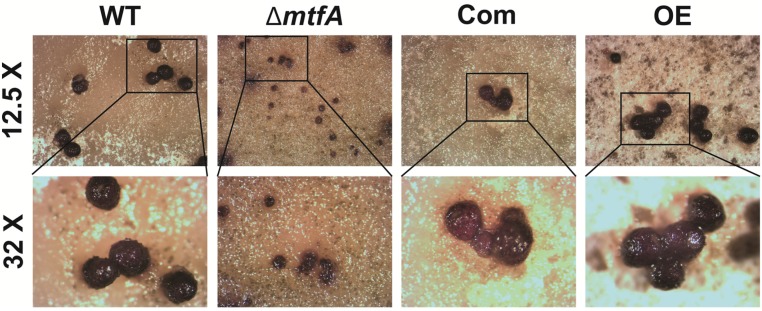
*mtfA* is necessary for the normal size of sclerotia. Micrographs (12.5× and 32× magnification) of YGT-top agar inoculated cultures after 14 days of incubation. Cultures were washed with 70% EtOH to improve the visualization of sclerotia.

In order to determine if *mtfA* plays a role-regulating conidiation, the strains were grown on top-agar inoculated cultures and in liquid stationary cultures. An increase in conidial production was observed in the Δ*mtfA* strain when compared to the isogenic controls in both types of cultures, whereas the OE*mtfA* strain exhibited a statistically-significant decrease in conidial production (Figure 4). This result was concomitant with the expression pattern of *brlA*, a key gene in the central regulatory pathway known to regulate conidiation in *Aspergillus* [39,40]. In our experiment, *brlA* transcription levels were significantly higher in the Δ*mtfA* strain at both time points measured, whereas they were reduced in the OE*mtfA* strain compared to the controls (Figure 4C). *A. flavus* also differentiates forming resistant structures termed sclerotia. Top-agar inoculated cultures were examined after 14 days of incubation. All of the strains were able to produce sclerotia under the experimental conditions assayed; however, the size of these structures was notably smaller in the Δ*mtfA* mutant with respect to the controls (Figure 5).

### 3.3. mtfA Is Necessary for Normal Virulence of A. flavus Peanut Infections

**Figure 6:**
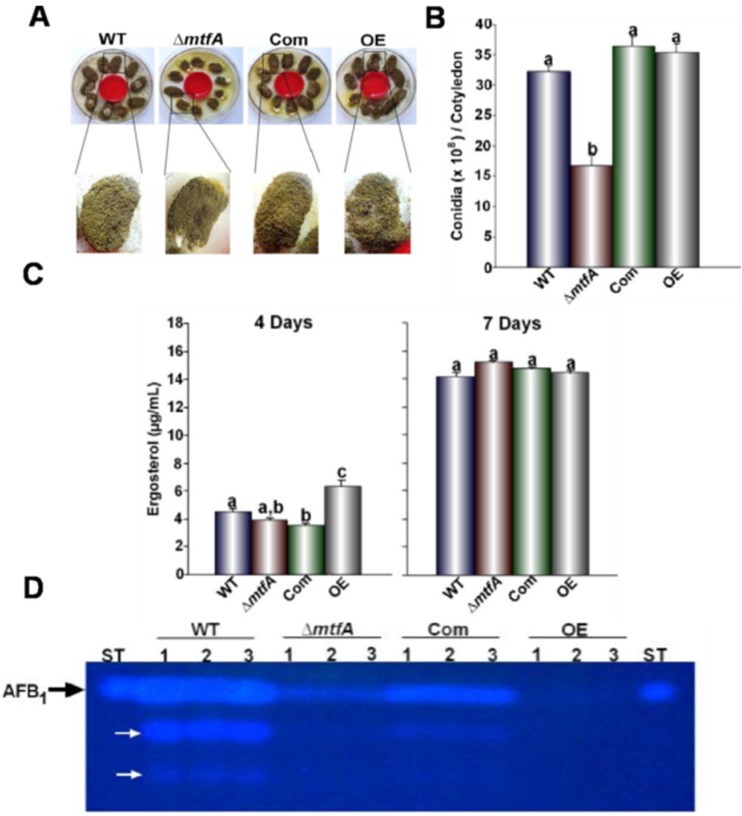
Role of *mtfA* in the *A. flavus* pathogenicity of peanut seeds. (**A**) Photographs of the NC94022 viable peanut line infected with the *A. flavus* strains after seven days of incubation. (**B**) Quantification of conidia present in infected peanut cotyledons in (**A**). (**C**) Ergosterol content in infected seeds after four and seven days of incubation. (**D**) TLC analysis of AFB_1_ levels present in infected peanut cotyledons. White arrows indicate unknown secondary metabolites. Error bars represent the standard error. Different letters above the bars represent significantly different values (*p* ≤ 0.05).

*Aspergillus flavus* is widely known as an opportunistic pathogen of economically-important oil seed crops. *mtfA* was previously shown to be necessary for virulence in *A. fumigatus* using an animal infection model [22]. However, the importance of *mtfA* in plant tissue colonization and mycotoxin contamination of this substrate has not yet been investigated. In our experiment, surfaced-sterilized viable peanuts seeds were inoculated with *A. flavus* wild-type, Δ*mtfA*, complementation and OE*mtfA* strains. Our results revealed a significant decrease in conidiation in the Δ*mtfA* infected seeds after seven days of incubation with respect to the wild-type levels (Figure 6A,B). We also examined ergosterol content in infected seeds as an indicator of fungal load. Although conidiation was reduced in the absence of *mtfA*, levels of fungal biomass were similar in the *mtfA* deletion mutant with respect to the controls. Interestingly, ergosterol levels also indicated that overexpression of *mtfA* resulted in faster colonization of seed tissue compared to the wild-type (Figure 6C). This was accompanied by a more notable softening of the seeds colonized by the OE*mtfA* strain compared to seeds infected by the other *A. flavus* strains tested (data not shown).

AFB_1_ content of infected peanuts seeds was also analyzed (Figure 6D). Importantly, a decrease in AFB_1_ levels was observed in seeds infected with the Δ*mtfA* and OE*mtfA* strains with respect to those infected with the isogenic controls. This reduction in AFB1 production was more pronounced when *mtfA* was overexpressed.

### 3.4. mtfA Positively Affects Lipase and Protease Activity While It Is Dispensable for Normal Amylase Activity

**Figure 7:**
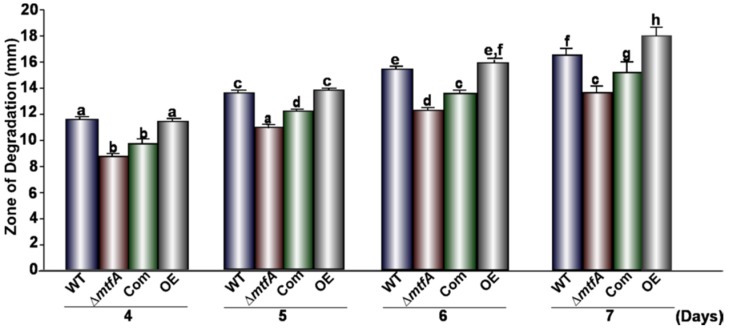
*mtfA* is necessary for normal lipase activity in *A. flavus*. The wild-type, Δ*mtfA*, complementation and overexpression strains were grown on tributyrin agar where zones of degradation were measured (mm) after 4, 5, 6 and 7 days of incubation. Error bars represent the standard error. The experiment was carried out with six replicates. Different letters above the bars represent significantly different values (*p* ≤ 0.05).

**Figure 8:**
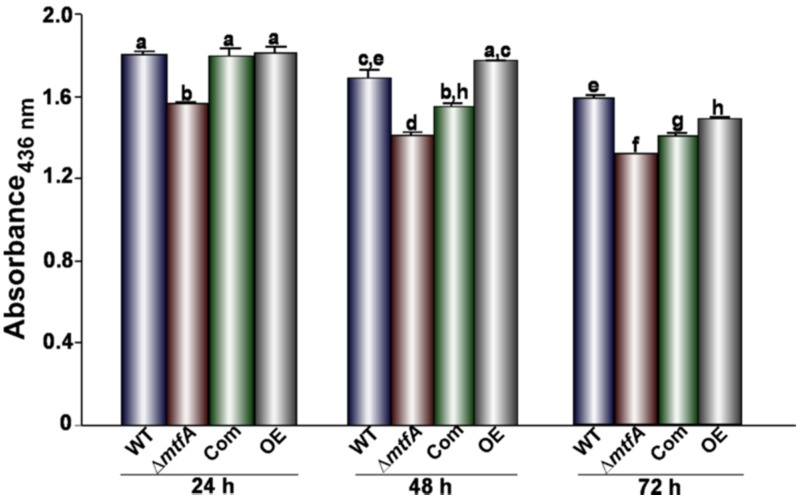
Effect of *mtfA* on protease activity. The *A. flavus* strains were grown in PMS broth at 37 °C and 250 rpm for 24 h before being shifted into liquid 0.1% GMM containing 8 mg/mL of BSA. Fungal supernatants were collected after 24, 48 and 72 h of incubation at 30 °C and at 250 rpm. Protease production was evaluated via an azocasein assay. Absorbance was read at 436 nm. The experiment was carried out with three replicates. Error bars indicate the standard error. Different letters above the bars represent significantly different values (*p* ≤ 0.05).

**Figure 9:**
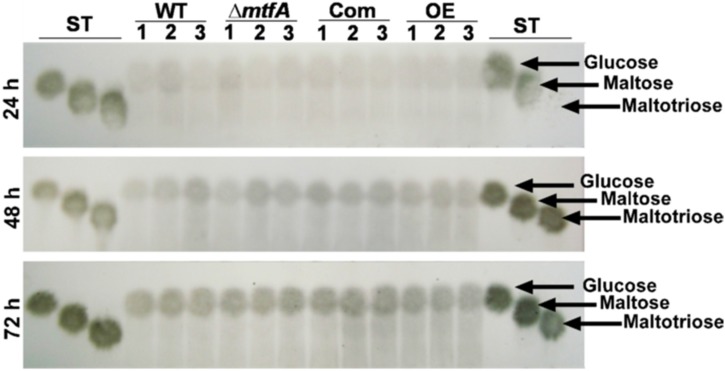
*mtfA* is dispensable for amylase production in *A. flavus*. The wild-type, Δ*mtfA*, complementation and overexpression strains were grown in PMS broth at 37 °C and 250 rpm for 24 h before being shifted into minimum medium containing 1% starch as the carbon source and incubated at 250 rpm. Fungal supernatants were collected after 24, 48 and 72 h of incubation at 30 °C, and amylase production was analyzed by using maltoheptose as the substrate. TLC analysis of degradation products was performed using glucose, maltose and maltotriose standards (ST) as the reference. The experiment was carried out with three replicates.

Due to the effect observed by *mtfA* on *A. flavus* virulence on peanut seeds, we investigated whether this regulator mediates hydrolytic activity that could be associated with seed colonization by this fungus. We first evaluated whether lipase activity is *mtfA*-dependent. The wild-type, Δ*mtfA*, complementation and OE*mtfA* strains were grown on a medium containing the short chain fatty acid tributyrin. Our analysis revealed a slight, but statistically-significant decrease in the zones of degradation in the Δ*mtfA* culture with respect to the controls at all time points assayed, indicating a reduction in lipase activity in the absence of *mtfA* (Figure 7). In addition, an increase in lipase activity was observed after seven days of incubation in the OE*mtfA* strain. Protease activity was also assessed using bovine serum albumin as the substrate. In this experiment, protease activity was also slightly downregulated in the absence of *mtfA* (Figure 8). We also analyzed the effect of *mtfA* on amylase activity in *A. flavus.* Our results indicated that this enzymatic activity is not affected by this regulatory gene (Figure 9).

## 4. Discussion

*Aspergillus flavus* is able to colonize economically-important crops, such as peanut, maize, cotton, tree nut and other oil seed crops. This fungus produces numerous secondary metabolites, including the carcinogenic and mutagenic compounds called aflatoxins (AFs). While consumption of AF-contaminated foods and feeds can have serious adverse health consequences in developing countries, in developed countries, it results mainly in adverse economic effects on producers; in the USA alone, *A. flavus* contamination of crops results in more than a billion dollars in losses per year [41]. Current methods are insufficient to control the severe detrimental effects caused by AF contamination. This has led to efforts to identify novel genes that regulate AF biosynthesis in *A. flavus* that can in turn serve as targets in strategies designed to control AF contamination by this mycotoxigenic fungus. Our current study revealed that AFB_1_ production is regulated by *mtfA* in *A. flavus*. The *mtfA* gene encodes a putative C_2_H_2_ zinc finger domain-type transcription factor first described in the model fungus *A. nidulans* [20]. Interestingly, as in the case of ST in *A. nidulans*, wild-type levels of *mtfA* expression are required to maintain normal AF biosynthesis. Both deletion and forced overexpression of *mtfA* led to a decrease in AF levels compared to the controls in *A. flavus*-infected seeds. The decrease in AF accumulation was more dramatic when this gene was overexpressed; in this case, AF was only present in trace amounts or absent. The negative effect of *mtfA* overexpression on AF production was also observed when the fungus was growing in a laboratory medium, where this decrease coincided with a reduction in the expression of *aflR*, encoding a transcription factor that acts as a specific AF cluster activator [14,15,16,17,18,19]. Reduction of *aflR* expression led to a reduction in the expression of the AF gene cluster, as indicated by the decrease in the expression of the structural gene *ver1*, used as a marker of cluster activation. The effect of the *mtfA* deletion on AF appears to be substrate-dependent, since Δ*mtfA* showed similar levels of AF as the wild-type when growing on YGT medium, while a decrease was observed when *A. flavus* was growing on seed. This decrease in toxin production caused by the absence of *mtfA* was also observed when ST was analyzed in the *A. nidulans mtfA* deletion mutant on glucose minimum medium [20]. Furthermore, the remarkably strong repressing effect of *mtfA* overexpression on toxin production was common in both fungi in all culture conditions assayed. Although alterations in *mtfA* expression did not prevent fungal colonization of the seeds, the resulting dramatic decrease or blockage of AFB_1_ production could be relevant for the development of new control methodologies to prevent AF contamination of crops.

Interestingly, our study also indicated that in addition to AF, the synthesis of many other compounds may also be affected by *mtfA*, indicating a broader *mtfA* regulatory impact on *A. flavus* secondary metabolism. This broader regulatory scope is conserved in other *Aspergillus* species. In *A. nidulans*, besides ST, *mtfA* regulates the synthesis of Penicillin and Terriquinone A [20]. Furthermore, recent transcriptome analyses of *mtfA* in *A. nidulans* and *A. fumigatus* revealed hundreds of genes under *mtfA* regulation, including secondary metabolite gene clusters [21]. It is likely that *mtfA* might have a similar effect on the *A. flavus* genome, governing the activation of other gene clusters, besides the AF cluster, directing the synthesis of multiple compounds, as indicated by our chemical analyses of both laboratory medium and infected seeds.

Fungal secondary metabolism has been shown to be genetically linked to morphological development [42]. As in the case of *A. nidulans* and *A. fumigatus*, the absence of *mtfA* in *A. flavus* affected conidiation; however, the effects observed on asexual development vary in different *Aspergillus* species. In the model fungus *A. nidulans*, deletion of *mtfA* resulted in a strain with reduced conidial production [20]. This was also observed in *A. flavus* Δ*mtfA* when growing on live seeds; however, when growing on synthetic culture medium colonies, hyper-conidiation, accompanied by an increase in the expression of *brlA*, a gene essential for conidiophore formation, was observed [39,40]. Overexpression of *mtfA* led to a significant decrease in *brlA* transcription and conidial production on laboratory medium, as was also observed in *A. fumigatus* [22], whereas this effect was not detected when *A. flavus* was growing on seeds. This suggests that *mtfA* regulation of conidiation is responsive to environmental conditions in *A. flavus*, and it varies in different fungal species. This species-dependent variation of the *mtfA* regulatory output in developmental events is in agreement with our previous *mtfA* comparative transcriptome analysis of *A. nidulans* and *A. fumigatus* [21], in which this divergent regulation of *mtfA* in development was noted, suggesting that certain regulatory circuit rewiring has occurred through evolutionary processes.

Previously, *mtfA* was shown to be necessary for cleistothecial production in *A. nidulans* [20]. Sclerotia have been shown to be vestigial structures of cleistothecia [43,44,45], and in some cases, ascospore-bearing ascocarps embedded within sclerotia (termed stromata) of *A. flavus* and *A. parasiticus* have been found [46,47]. This common origin between cleistothecia and sclerotia suggests that conserved genetic regulatory pathways controlling cleistothecial formation could also affect sclerotial production. Our study showed that sclerotial formation was also influenced by *mtfA*. Although sclerotia were produced in the absence of *mtfA*, their size was reduced in comparison to the wild-type. It is possible that this size reduction could affect the survival of these resistance structures under adverse conditions in the field.

During crop colonization, *A. flavus* produces a variety of extracellular hydrolytic enzymes to obtain nutrients from the host [17,35,36,38,48]. For this reason, we also examined whether *mtfA* has a role in regulating lipase, amylase and protease activity in the secretome. Our results indicated that *mtfA* has a minor effect on these activities; only a small reduction in lipase and protease activity was observed in the absence of this transcription factor. This coincided with similar colonization levels by all of the strains tested, as indicated by ergosterol levels measured in infected seeds. A slight increase in lipase activity was detected over time in the OE*mtfA* strain with respect to the wild-type. It is possible that this could be associated with the fact that seeds colonized by the *A. flavus* strain overexpressing *mtfA* also showed a slightly faster pace in seed colonization and maceration compared to those infected with the wild-type.

In conclusion, our studies indicated that *mtfA* affects several cellular processes in the agriculturally-important fungus *A. flavus*, influencing development in response to the environment, and in this way, affecting dissemination and survival of this fungal species. However, the most relevant finding in this study is the drastic decrease or suppression of AF production on infected live seeds achieved by altering the expression pattern of the *mtfA* gene, making *mtfA* a desirable candidate to target in control strategies against AF contamination of crops by *A. flavus*. In addition, we showed that *mtfA* not only affects AF production in this fungus, but it also appears to govern the synthesis of other secondary metabolites. This is the first study of *mtfA* homologs in an opportunistic plant pathogen. However, the fact the *mtfA* is conserved in numerous Ascomycetes, together with its broad effect on secondary metabolism observed in *Aspergillus* species, suggests that an *mtfA*-based control approach could also be used to reduce the detrimental effects of other mycotoxigenic fungi, including other plant pathogens that endanger our food commodities.
